# The long noncoding RNA HOTAIR activates the Hippo pathway by directly binding to SAV1 in renal cell carcinoma

**DOI:** 10.18632/oncotarget.17414

**Published:** 2017-04-25

**Authors:** Guanghui Hu, Binbin Dong, Jingwei Zhang, Wei Zhai, Tiancheng Xie, Bisheng Huang, Chi Huang, Xudong Yao, Junhua Zheng, Jianping Che, Yun-Fei Xu

**Affiliations:** ^1^ Department of Urology, Shanghai Tenth People's Hospital, Tongji University, Shanghai, China; ^2^ Department of Urology, Yancheng Third People's Hospital, Yancheng, Jiangsu, China; ^3^ Department of Urology, Renji Hospital, Shanghai Jiaotong University, Shanghai, China; ^4^ Department of Urology, Shanghai Tenth People's Hospital, Nanjing Medical University, Nanjing, Jiangsu, China

**Keywords:** renal cell carcinoma, HOTAIR, hippo pathway, SAV1

## Abstract

The long noncoding RNA HOTAIR promotes the development and progression of several tumors. Here, the clinical significance and role of HOTAIR in renal cell carcinoma (RCC) tumorigenesis were explored. The results showed that increased expression of HOTAIR predicted a poor prognosis of RCC after surgery. HOTAIR promoted RCC cell proliferation and growth *in vitro* and *in vivo*. The expressions of HOTAIR and Salvador homolog 1 (SAV1) were inversely correlated in clinical RCC samples. HOTAIR downregulated SAV1 by directly binding to the SAV1 protein and enhanced histone H3K27 methylation. Loss of function of SAV1 activated the Hippo pathway. HOTAIR could be a potential therapeutic target in RCC.

## INTRODUCTION

Renal cell carcinoma (RCC), which affects nearly 300,000 people worldwide annually, is the ninth most commonly diagnosed cancer in men worldwide and causes approximately 100,000 deaths each year [1]. Nephrectomy (or partial nephrectomy) is the standard surgical therapy for primary localized RCC [2]. However, in cases of metastasis or recurrence, targeted therapy is the treatment of choice because of the intrinsic resistance to conventional chemotherapy and radiotherapy. Currently, there are several targeted drugs for RCC including tyrosine kinase inhibitors (TKIs), antibodies against vascular endothelial growth factor, and mTOR pathway inhibitors [3, 4]. However, these drugs do not improve overall survival and progression-free survival in all cases, and some patients show inherent resistance or acquire resistance after 6–12 months of treatment [3]. Therefore, the development of novel targeted drugs is important.

Long noncoding RNAs (lncRNAs) are noncoding transcripts of >200 nt in length. LncRNAs are involved in many human diseases including cancer, and they play a significant role in tumorigenesis and cancer progression [5, 6]. The lncRNA HOTAIR was initially found be involved in primary breast cancer and breast cancer metastasis [7]. Clinical studies demonstrated that HOTAIR overexpression is a predictor of tumor progression and overall survival in patients with diverse types of cancer [8–10]. Frequent HOTAIR upregulation is associated with the malignant behavior of non-small cell lung cancer [9]. HOTAIR interacts with polycomb repressive complex 2 (PRC2), leading to the hypermethylation of histone H3 lysine 27 (H3K27) and aberrant gene expression. HOTAIR activates the Wnt/β-catenin signaling pathway by promoting histone H3K27 methylation in the promoter region of WIF-1 [11].

Although the involvement of HOTAIR in tumor-igenesis has been reported extensively, its role in the development and progression of RCC and the underlying mechanisms have not been clearly demonstrated. Here, we examined the prognostic value of HOTAIR in RCC and showed that altered HOTAIR expression repressed the transcription of Salvador homolog 1 (SAV1), leading to the activation of the Hippo pathway. The present study is the first to show that HOTAIR directly regulates RCC development and progression by activating the Hippo pathway.

## RESULTS

### Increased expression of HOTAIR correlates with RCC patient prognosis

**Figure 1:**
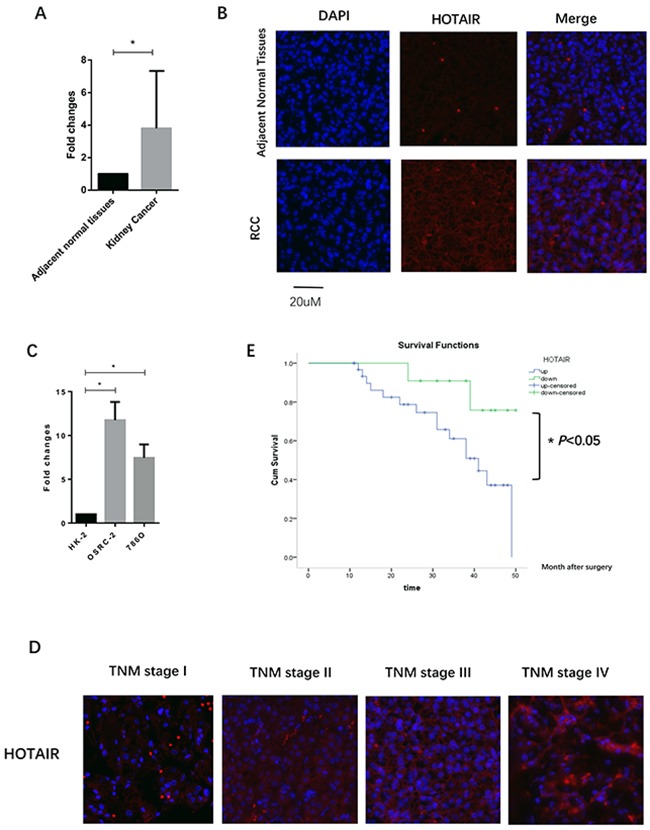
HOTAIR is overexpressed in renal cell carcinoma (RCC) **(A and B)** Elevated expression of HOTAIR was detected in RCC clinical samples by quantitative RT-PCR **(A)** and lncRNA FISH **(B)**. **(C)** Expression of HOTAIR in RCC cell lines (786O and OSRC-2) compared with the normal kidney renal tubular epithelial cell line HK2. **(D)** Expression of HOTAIR was increased in high stage RCC (III and IV), as compared with stage I and II. **(E)** Patients with high expression of HOTAIR had a short overall survival after surgery.

HOTAIR expression in clinical RCC samples was examined by qRT-PCR and RNA fluorescence in situ hybridization (FISH). HOTAIR was overexpressed in 32 of 43 (74.4%) patients (*P* < 0.0001, Figure 1A and 1B). HOTAIR was overexpressed in 786O and OSRC-2 cells compared with normal HK2 cells (786O vs. HK2, *P* < 0.05; OSRC-2 vs. HK2, *P* < 0.05, Figure 1C). Forty-three patients were included and classified into two groups: high HOTAIR group (HOTAIR expression fold changes ≥ median fold changes, n=32) and low HOTAIR group (HOTAIR expression fold changes < median fold changes, n=11). Clinicopathological factors were compared between the two groups (Table 1). HOTAIR was markedly upregulated in higher stage RCC, as detected by FISH (Figure 1D). Elevated expression of HOTAIR in clinical samples was correlated with T stage (*P* < 0.001, Table 1) and lymph node metastasis (*P* < 0.001, Table 1). However, HOTAIR overexpression was not associated with patient age and gender. Kaplan-Meier survival curves showed that patients with high expression levels of HOTAIR (n=32) had shorter overall survival than those with low expression levels of HOTAIR (n=11, *P* = 0.042, log rank test; Figure 1E). These results indicated that HOTAIR may be an independent prognostic factor in RCC and may promote RCC progression and development.

**Table 1:** HOTAIR expression and clinicopathological characteristics in clear cell renal cell carcinoma

		Higher expression of HOTAIR (n=32)	Lower expression of HOTAIR(n=11)	*P* value
Age		54.56±12.90	60.09±8.93	0.129
Gender	Male	17	6	
	Female	15	5	
T stage	1+2	11	7	0.037*
	3+4	21	4	
N	N0	11	8	0.038*
	N1	21	3	
Meta	M0	18	9	0.166
	M1	14	2	
Sexual	Male	17	6	0.935
	Female	15	5	
Location	Left	16	3	0.294
	Right	16	8	
Recurrence	Yes	20	2	0.016
	No	12	9	
Survival	Yes	17	9	0.154
	No	15	2	

### HOTAIR promotes cell proliferation and migration of RCC cells

**Figure 2:**
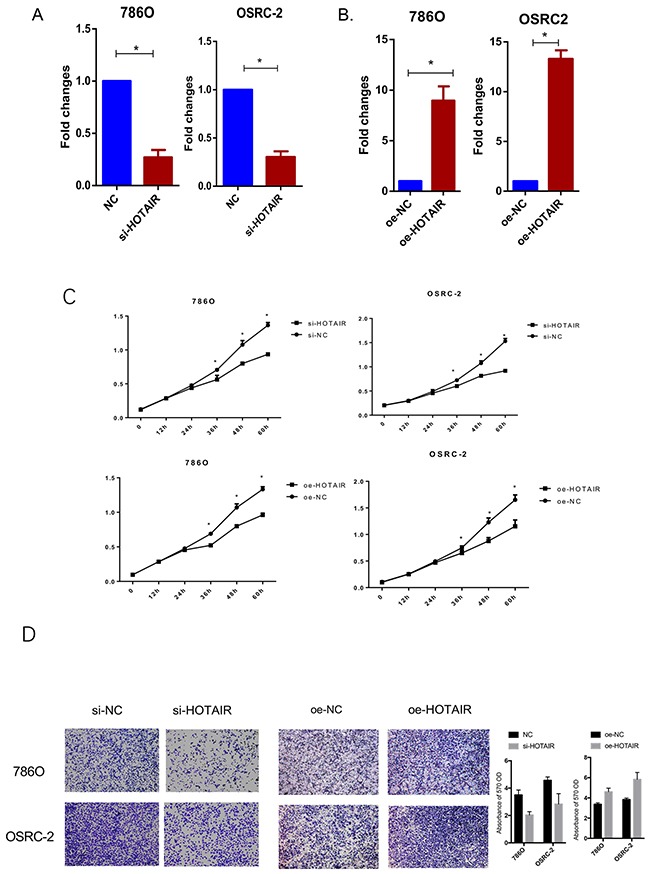
Knockdown and overexpression of HOTAIR affect RCC cell proliferation and migration **(A)** Expression of HOTAIR was successfully knocked down. **(B)** Overexpression lentivirus was successfully constructed and transfected into 786O and OSRC-2 cells. **(C)** HOTAIR knockdown significantly suppressed RCC cell proliferation, whereas HOTAIR overexpression promoted cell proliferation. **(D)** Cell migration was inhibited by HOTAIR knockdown and enhanced by HOTAIR overexpression.

HOTAIR knockdown and overexpression lentivirus constructs (Figures 2A and 2B) were generated to assess the effects of HOTAIR on cell proliferation and migration of RCC cell lines. As shown in Figure 2C, RCC cell proliferation was suppressed by HOTAIR silencing, whereas it was enhanced by HOTAIR overexpression, suggesting that HOTAIR promoted RCC cell proliferation. Migration activity was reduced in si-HOTAIR infected RCC cells, whereas HOTAIR overexpression increased RCC cell migration (Figure 2D). These findings indicated that HOTAIR promotes RCC cell proliferation and migration.

### Negative correlation between HOTAIR and SAV1

**Figure 3:**
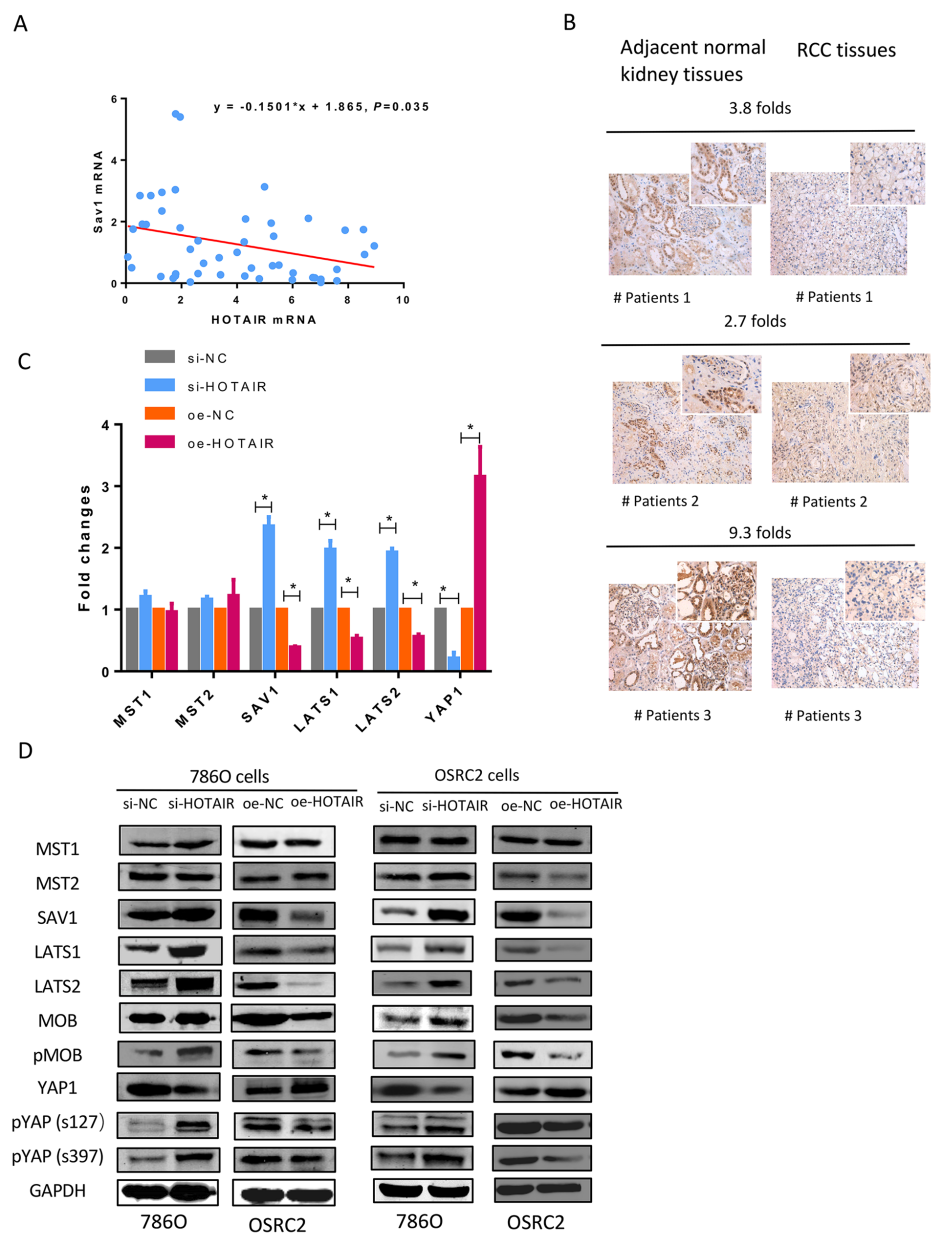
Negative correlation between HOTAIR and SAV1 **(A)** HOTAIR expression was negatively correlated with SAV1 at the mRNA level in RCC clinical samples. **(B)** SAV1 protein was downregulated in cancer tissues with high HOTAIR expression levels. **(C and D)** HOTAIR knockdown significantly downregulated SAV1 mRNA and protein, whereas HOTAIR overexpression upregulated SAV1. Hippo pathway proteins were detected with a Hippo Pathway Antibody Kit (cat:8579, Cell Signal Technology, Beverly, MA, USA). SAV1, large tumor suppressor homolog (LATS)-1, LATS2, phosphorylated MOB, Yes-associated protein 1 (YAP1), and phosphorylated YAP1 levels were significantly altered by HOTAIR knockdown or overexpression.

A negative association between HOTAIR and SAV1 mRNA and protein was observed in clinical RCC specimens (Figures 3A and 3B). SAV1 was significantly downregulated in tissues showing HOTAIR overexpression compared with adjacent normal kidney tissues. HOTAIR knockdown upregulated SAV1 protein expression, whereas HOTAIR overexpression downregulated SAV1 (Figure 3D).

### HOTAIR directly binds to the SAV1 protein

**Figure 4:**
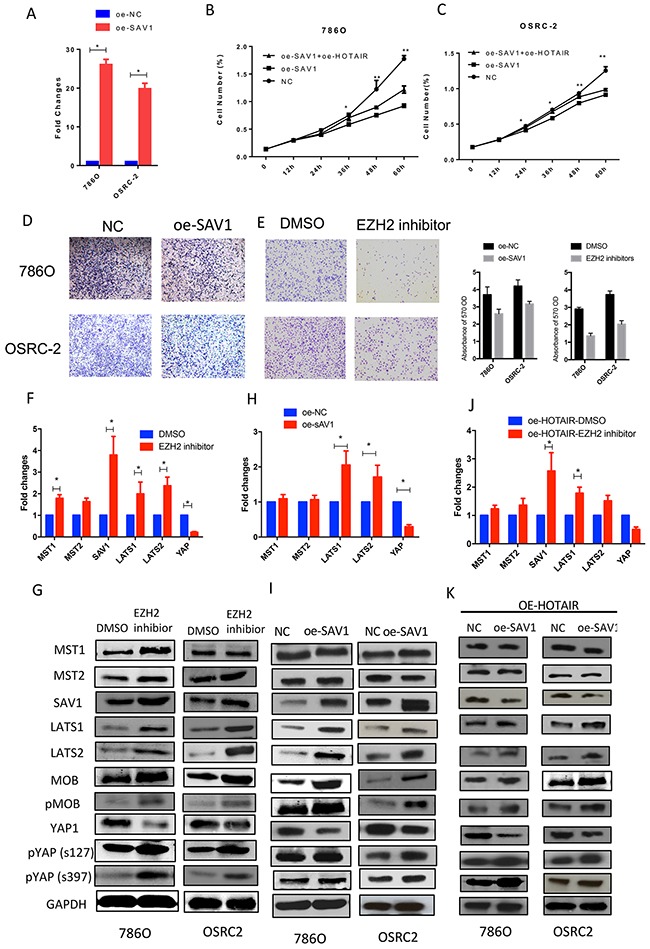
HOTAIR binds to SAV1 and activates the Hippo pathway **(A)** Restoration of SAV1 expression in 786O and OSRC2 cells. **(B and C)** Restoring SAV1 expression or inhibiting EZH2 attenuated the effect of HOTAIR on promoting cell proliferation. **(D and E)** SAV1 overexpression inhibited the migration of 786O and OSRC2 cells, similar to treatment with GSK503. **(F, G, H and I)** Hippo pathway activation was reversed by restoration of SAV1 or GSK503 treatment in 786O and OSRC2 cells. **(J, and K)** Restoration of SAV1 and treatment with EZH2 inhibitor inactivated the Hippo pathway.

**Figure 5:**
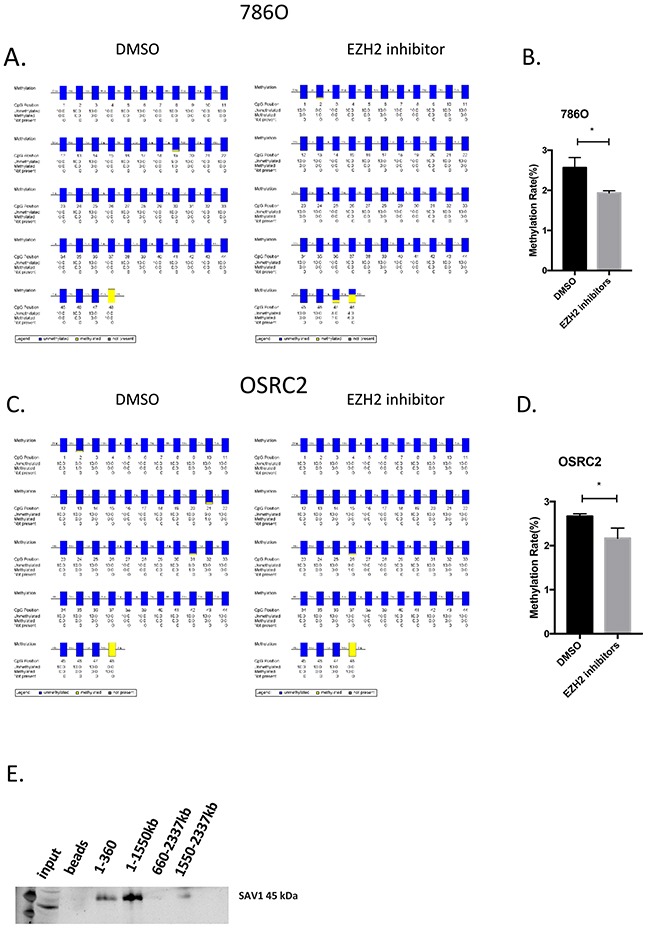
HOTAIR directly binds to SAV1 **(A–D)** Methylation status and rate of SAV1 CpG island in 786O and OSRC2 cells. **(E)** RNA-pulldown revealed that HOTAIR binds to SAV1.

As SAV1 was downregulated in RCC samples, a SAV1 overexpression plasmid was constructed to restore its expression in RCC cells (Figure 4A). Restoration of SAV1 expression in RCC cells inhibited RCC cell proliferation and migration (Figure 4B, 4C and 4D). As shown in Figure 3A and 3B, HOTAIR expression was negatively related to SAV1 mRNA and protein expression. HOTAIR binds to the PRC2 complex, which functions in the methylation of many genes. Treatment of RCC cells with an EZH2 inhibitor (GSK503) upregulated modulators of the Hippo pathway and SAV1 expression at the mRNA and protein levels (Figures 4F and 4G). Overexpression of SAV1 in RCC cells inactivated the Hippo pathway similar to the effect of treatment with the EZH2 inhibitor (Figure 4H, 4I, 4J and 4K). To confirm that HOTAIR suppresses SAV1 expression by modulating EZH2 methylation activity, the methylation level of the SAV1 promoter region was detected after treatment with GSK503. However, as shown in Figure 5A and 5C, the methylation activity of the SAV1 promoter region was only slightly suppressed by the EZH2 inhibitor in 786O and OSRC2 cells. An RNA pull-down assay showed that HOTAIR directly binds to the SAV1 protein (Figure 5E).

### HOTAIR promotes Yes-associated protein 1 (YAP1) translocation

**Figure 6:**
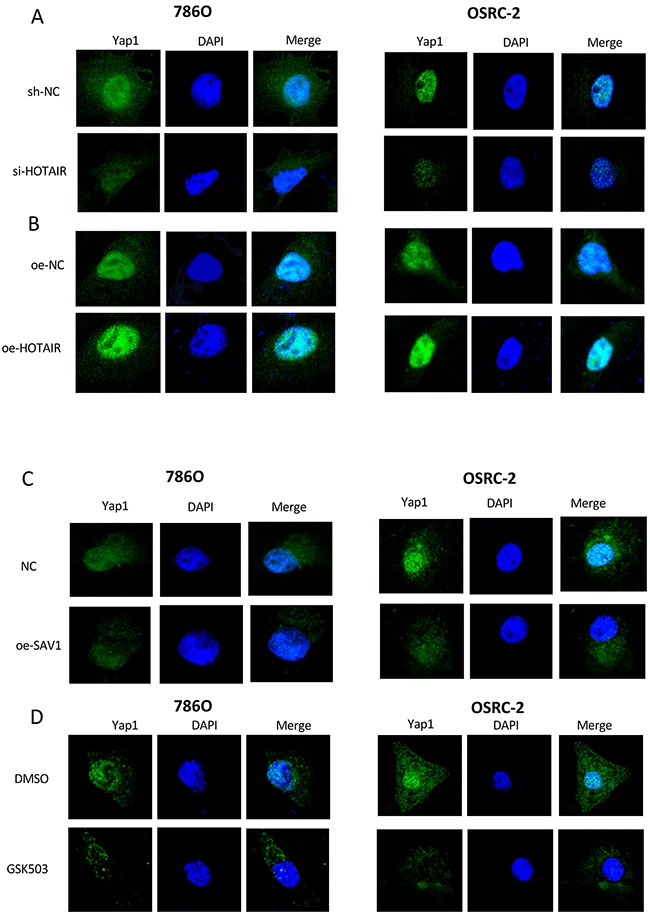
HOTAIR promotes the nuclear translocation of YAP1 **(A)** HOTAIR knockdown inhibited YAP1 translocation. **(B)** HOTAIR overexpression significantly upregulated YAP1 expression and nuclear translocation. **(C and D)** SAV1 overexpression or EZH2 inhibitor (GSK503) suppressed YAP1 expression and nuclear translocation.

To determine whether HOTAIR activated the Hippo pathway, the location of YAP1 was assessed after overexpression or knockdown of HOTAIR, restoration of SAV1, and GSK503 treatment (Figure 6). Immunofluorescence analysis showed that HOTAIR knockdown inhibited the nuclear translocation of YAP1 in RCC cells (Figure 6A). Both EZH2 inhibitor and restoration of SAV1 had the same effect as HOTAIR knockdown (Figures 6C and 6D). HOTAIR overexpression promoted YAP1 translocation to the nucleus (Figure 6B). These results indicated that HOTAIR activated the Hippo pathway.

### HOTAIR promoted tumor growth *in vivo*

**Figure 7:**
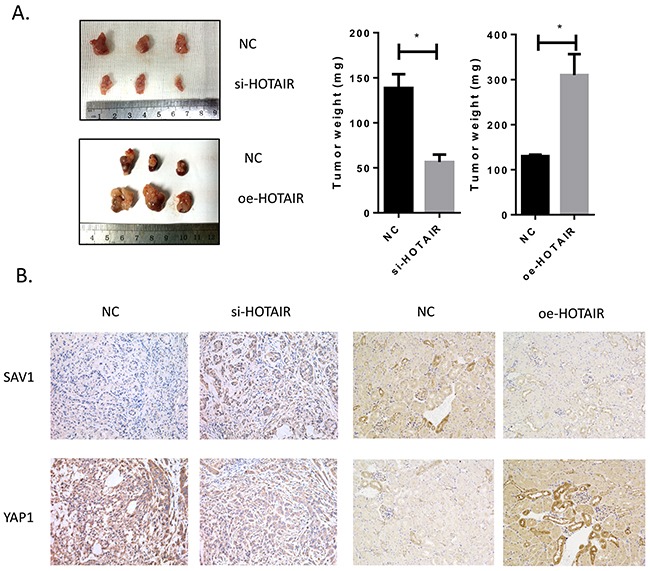
Tumor growth was suppressed by HOTAIR knockdown **(A)** HOTAIR knockdown attenuated tumor growth *in vivo*. **(B)** After harvesting tumors, SAV1 and YAP1 were detected by immunohistochemistry in control and treated groups. The results were consistent with *in vitro* findings.

The role of HOTAIR in RCC cancer growth was examined *in vivo*. HOTAIR knockdown significantly inhibited tumor growth, whereas HOTAIR overexpression promoted tumor growth (Figure 7A). The results of immunohistochemical detection of key modulators (SAV1 and YAP1 protein) in tumors were consistent with *in vitro* findings (Figure 7B), confirming that HOTAIR promotes RCC development and growth by activating the Hippo pathway through direct binding to the tumor suppressor SAV1.

## DISCUSSION

**Figure 8:**
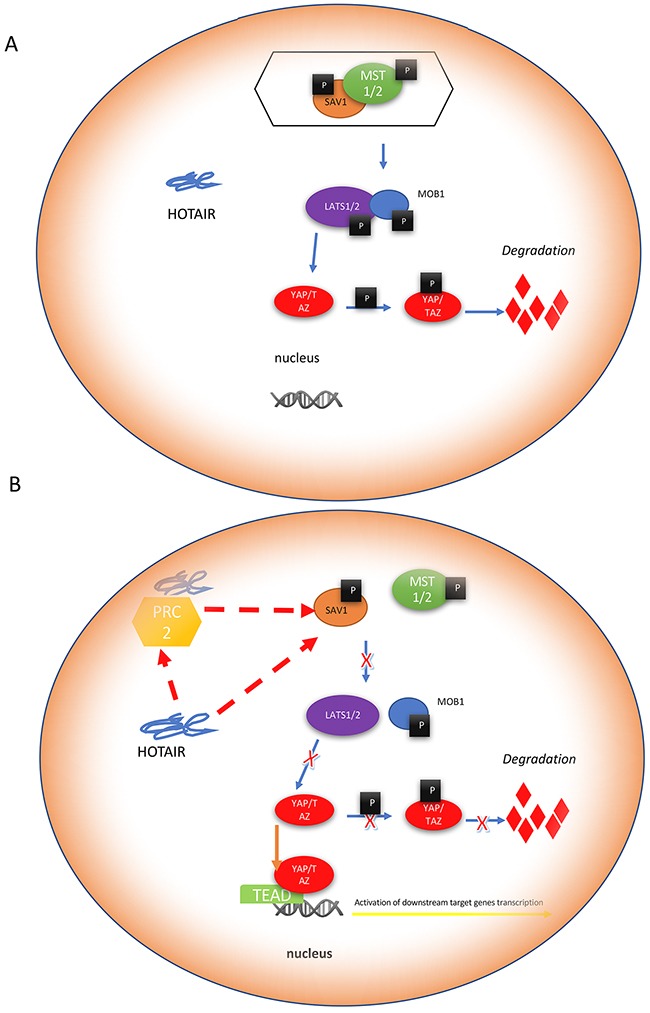
Mechanism of Hippo pathway regulation by HOTAIR HOTAIR activates the Hippo pathway by directly binding to the upstream regulator scaffolding protein SAV1. **(A)** SAV1 interacts with phosphorylated mammalian sterile 20-like kinase (MST)1/2 to form a complex, which activates LATS1/2 kinases by phosphorylation. Activated LATS1/2, in combination with their co-activator MOB1, phosphorylate YAP/TAZ on Ser127. Phosphorylated YAP at Ser127 creates a 14-3-3 binding site in an Akt-dependent manner and translocates to the cytoplasm. Cytoplasmic retention of YAP/TAZ results in its ubiquitination by the β-TrCP (SCF) ubiquitin ligase complex, which is mediated by phosphorylation of YAP on Ser397. **(B)** HOTAIR binds to SAV1, which inhibits its interaction with MST1/2. The subsequent phosphorylation and activation of LATS1/2 cannot be completed without activation of SAV1. These activities cause the failed phosphorylation of YAP on Ser127. In addition, phosphorylation on Ser397 is also inhibited by HOTAIR. In the absence of these phosphorylation events, cytoplasmic YAP cannot be degraded and is translocated to the nucleus, which can result in the transcription of specific target genes involved in cell proliferation and apoptosis.

In the present study, we showed that increased expression of HOTAIR in RCC was associated with tumor-lymph node-metastasis (TNM) stage and inversely correlated with prognosis. HOTAIR promoted RCC cell proliferation and migration by activating the Hippo pathway (Figure 8). HOTAIR could directly bind to SAV1, a negative regulator of the Hippo pathway.

Increasing evidence suggests that the lncRNA HOTAIR acts as an oncogene, promoting the development and progression of various types of cancer [12–18]. HOTAIR overexpression promotes breast cancer cell proliferation, whereas its depletion significantly impairs cell survival and abolishes tamoxifen-resistant cell growth [7]. A recent study showed that HOTAIR is overexpressed in 96.15% (75/78) of esophageal squamous cell carcinoma patients [19]. Patients with high expression of HOTAIR have a short 5-year survival rate [19]. The present study showed that HOTAIR expression was higher in RCC tissues than in adjacent normal kidney tissues. HOTAIR expression was significantly correlated with TNM stage, lymph node metastasis, and shorter patient survival after surgery. Zhang et al. reported that HOTAIR competitively binds to the N-terminal domain of the AR protein, preventing AR degradation by inhibiting its ubiquitination. Depletion of HOTAIR in enzalutamide-resistant PCa cells results in the recovery of their sensitivity to enzalutamide and decreased tumor growth [20].

HOTAIR interacts with PRC2, modulating chromatin structure and DNA methylation [21, 22]. It is required for gene silencing of the HOXD locus by PRC2, which controls the expression of several critical tumor suppressors such as WIF-1, SETD2, and PTEN [11, 23]. We showed that HOTAIR directly binds to SAV1 and activates the Hippo pathway. In mammals, the Hippo pathway consists of several important components including the scaffolding protein SAV1, the upstream serine/threonine kinases (Ser/Thr) mammalian sterile 20-like kinase 1 (MST1) and MST2, the Ser/Thr protein kinases large tumor suppressor homolog 1 (LATS1) and LATS2, which interact with Mob1, and the transcriptional co-activator YAP1 [24–27]. SAV1, which contains a C-terminal SAV–RASSF–Hippo (SARAH) domain, is required for MST1 and MST2 activation [28–30]. SAV1 forms a complex with MST1 and MST2. The SAV1/MST1/MST2 complex phosphorylates and activates LATS1/2 in complex with its regulatory protein MOB1, leading to the phosphorylation and inactivation of the YAP1 oncoprotein and TAZ.

SAV1 is downregulated in RCC, resulting in the activation of the Hippo pathway [28, 31]. SAV1 is significantly downregulated in high-grade clear cell RCCs because of copy number loss at 14q22.1, and its deficiency leads to enhanced proliferation of renal epithelial cells. However, in the present study, we showed that HOTAIR could directly regulate SAV1. Activation of the Hippo pathway promotes RCC cell proliferation and migration. The Hippo pathway drives cell proliferation and growth in various cancers [32–34]. YAP1 is a key transcription factor that controls the transcription of several oncogenes including EGFR and SOX9 [35, 36]. Recent studies showed that the Hippo pathway modulates cancer invasion, metastasis, drug resistance, and the maintenance of cancer cell stemness [33, 37–41]. The Hippo pathway is therefore a potential drug target in cancer therapy.

In conclusion, HOTAIR is an oncogene involved in RCC development and progression and a potential predictor of prognosis after surgery. It directly binds to SAV1 and suppresses its function, resulting in the activation of the Hippo pathway. HOTAIR may be a potential alternative drug target in RCC.

## MATERIALS AND METHODS

### Cell culture and transfection

Renal cancer cell lines (786O and OSRC2) and normal kidney epithelial cells (HK2) were purchased from the American Tissue Culture Collection (Rockville, MD, USA). The 786O and OSRC2 cells were grown in RP1640 medium (Hyclone, Logan, UT, USA) supplemented with 10% fetal bovine serum (Hyclone, 10270) and 1% penicillin/streptomycin (Invitrogen, Carlsbad, CA, USA; 15140). Renal cancer cells were transfected with HOTAIR shRNA-lentivirus and overexpression lentivirus. For EZH2 inhibitor treatment, the vehicle group was treated with 1% DMSO and the treatment group was cultured in medium with 5 μM GSK503 (MedChem Express, Monmouth Junction, NJ, USA).

### Lentivirus

Lentiviral vectors for the human HOTAIR-expressing sequence were constructed by Hanyin Co. (Shanghai, China). The primer sequences for human HOTAIR are listed in Supplementary Material and Methods. The recombinant HOTAIR-expressing lentivirus and the negative control lentivirus were prepared and titered to 10^8^ TU/ml (transfection unit).

### Clinical tissue specimens

A total of 43 patients (23 men and 20 women) with pathologically confirmed RCC were enrolled (Shanghai 10th People's Hospital, Shanghai, China). All patients had undergone radical nephrectomy between 2009 and 2015. All the tumors were stored in liquid nitrogen. The mean age was 56.1±12.2 years (Table 1). The patients were classified according to World Health Organization criteria and staged according to the TNM classification system. The pathologic type for all patients was clear cell RCC. Samples were obtained from the patients after obtaining signed informed consent in Shanghai 10th People's Hospital of Tongji University.

### RNA extraction and quantitative real time RT-PCR

RNA was isolated using the RNeasy mini kit (Qiagen, Hilden, Germany; Cat No.: 74104) according to the manufacturer's protocol. For mRNA analysis, RNA (500 ng) was reverse transcribed using random hexamers and Q-PCR was performed using the Applied Biosystems Prism 7900 Fast Sequence Detection System (Bio-Rad, Hercules, CA, USA). The 2-ΔΔCt method was used to quantify gene expression changes. Relative changes were normalized to GAPDH for differentiation experiments. The relative expression levels of HOTAIR and other mRNAs were measured using a two-step Kappa assay according to the manufacturer's protocol. RNA (10 ng) for miRNA analysis was reverse transcribed using the TAKARA Reverse Transcriptase Kit (Applied Biosystems, Carlsbad, CA, USA). The KAPA SYBR^®^ FAST qPCR kit was prepared using the Applied Biosystems Prism 7900 Fast Sequence Detection System (Applied Biosystems). The expression levels of HOTAIR were based on the amount of the target message relative to that of the GAPDH transcript as a control to normalize the initial input of total RNA.

### RNA fluorescence in situ hybridization

A probe for the human lncRNA HOTAIR (5′-TGCGT GGTTC GCTTT CACCT TCGTC TGG-3′) containing a biotin label was used for RNA FISH analysis. The methods and protocols were as previously described by Gupta et al. [42].

### Western blot analysis

Total cell protein (10–30 μg) was used for western blot analysis. Samples were resolved in 4–12.5% Precise Protein Gels and transferred to nitrocellulose membranes. The membranes were blocked in 5% fat-free milk in Tris-buffered saline (TBS) with 0.1% Tween 20 (TBST) for 1 h followed by incubation with primary polyclonal and monoclonal antibodies of the Hippo pathway kit (cat:8579, Cell Signal Technology, Beverly, MA, USA) and the internal control GAPDH (cat: 5174, Cell Signal Technology) overnight at 4°C. Blots were washed in TBST and labeled with horseradish peroxidase-conjugated secondary anti-rabbit antibody (Cell Signaling Technology). Membranes were processed by the LI-COR Odyssey Imaging System (LI-COR Biosciences, Lincoln, NE, USA).

### Immunohistochemical study

The antibodies used were the same as those used for western blotting. SAV1 and YAP1 expression was evaluated according to the intensity and extent of staining. The proportion of stained cells per specimen was semi-quantitatively evaluated and scored as follows: 0 for staining ≤1%; 1 for 2–25%; 2 for 26–50%; 3 for 51–75%; and 4 for >75% of the examined cells. The staining intensity was stratified as follows: 0, negative staining; 1, weak staining; 2, moderate staining; and 3, strong staining. The results were evaluated using the following formula: total score = proportion score × intensity score. A total score of 0–12 was graded as negative (-; score: 0–4) or positive (+; score: 5–12).

### *In vitro* migration assay

The cell migration assay was performed according to the guidelines provided by the Corning Transwell protocol (Corning, NY, USA; 3422). Briefly, 48 h post transfection cells were seeded in the upper chamber at a density of 1 × 10^5^ cells/ml in FBS 1% medium and allowed to invade the membrane under the effect of a chemoattractant (FBS 10%) added into the lower chamber. After 20 h at 37°C, non-invading cells were removed from the upper chamber by scrubbing with a cotton swab while invading cells were fixed to nm was detected using a Microplate Reader (BIOtek, Winooski, VT, USA) the bottom surface with 95% neutral buffered ethyl alcohol, then stained with Crystal Violet and counted by microscope. Images of the areas were acquired with a LEICA microscope (Leica, Wetzlar, Germany). Crystal Violet was washed with glacial acetic acid and absorbance at 573.

### RNA pulldown

T7 promoter tagged oligos were produced by PCR. Biotin-labeled HOTAIR RNA was *in vitro* transcribed with the Biotin RNA labeling mix (Roche, Indianapolis, IN, USA) and T7 RNA polymerase (Roche) and purified with the RNeasy MiniKit (Qiagen). RNAs were incubated with protein extract from 786O cells in the presence of anti-RNase and protease inhibitor cocktail. Proteins pulled down by biotinylated RNA were subjected to western blot analysis. The remaining process was the same as that used for western blotting.

### DNA methylation analysis

The methylation status of CpG motifs in the SAV1 region was determined using bisulfite genomic sequencing. gDNA was bisulfite-modified using a FAST Bisulfite kit (Qiagen, cat:59824). Amplification was performed using an AccuPrime PCR kit (Invitrogen) to amplify the bisulfite-modified DNA. Bisulfite-specific primer sequences were designed and are listed in Supplementary Materials. Amplicons were cloned according to the manufacturer's instructions, and subsequently, inserts were sequenced. Quality control of DNA methylation data was performed with BiQ Analyzer software (Max Planck Institute, Munich, Germany).

### Statistical analysis

The RT-PCR results were analyzed using the ΔΔCt method (Applied Biosystems User Bulletin no. 2). A Kaplan-Meier analysis model was used to evaluate the effect of HOTAIR on ccRCC overall survival. All statistical analyses were performed using SPSS 20.0 and Graphpad Prism 6.0 software.

## SUPPLEMENTARY MATERIALS AND METHODS


