# Supplementary material for: The long noncoding RNA HOTAIR activates the Hippo pathway by directly binding to SAV1 in renal cell carcinoma
Source: Oncotarget. 2017 Apr 25;8(35):58654–67. doi: 10.18632/oncotarget.17414 (PMC5601682; doi:10.18632/oncotarget.17414)
Supplement: Supplementary file 1 [file oncotarget-08-58654-s001.pdf]

The long noncoding RNA HOTAIR activates the Hippo pathway by directly binding to SAV1 in renal cell carcinoma

MATERIALS AND METHODS

Primer Sequence for Quantitative Real time Polymerase Chain Reaction

HOTAIR: 5'-GGTAGAAAAAGCAACCACGAAGC-3'
Reverse: 5'-ACATAAACCTCTGTCTGTGAGTGCC-3'
SAV1 5'-CTCTTGAGCGAGAAGGACTTCC-3'
Reverse: 5'-GAGGTACACTAGGAGCACAGG-3'
MST1 5'-CCTTGGTGCTACACAACAGAC-3'
Reverse: 5'-CAGACCTTGGTCGAGGAACTT-3'
MST2 5'-CTTTGGTCCGATGATTTCACCG-3'
Reverse: 5'-GGATGCTGTAAAAGTTGTGTTGC-3'
LATS1 5'-AATTTGGGACGCATCATAAAGCC-3'
Reverse: 5'-TCGTCGAGGATCTTGGTAACTC-3'
LATS2 5'-TGGCACCTACTCCCACAG-3'
Reverse: 5'-CCAAGGGCTTTCTTCATCT-3'
MOB1 5'-CAGCAGCCGCTCTTCTAAAAC-3'
Reverse: 5'-CCTCAGGCAACATAACAGCTTG-3'
YAP1 5'-CGCTCTTCAACGCCGTCA-3'
Reverse: 5'-AGTACTGGCCTGTCGGGAGT-3'
GAPDH 5'-GAGTCAACGGATTTGGTCGT-3'
Reverse: 5'-GACAAGCTTCCCGTTCTCAG-3'

Primer for Methylation Analysis
Forward: 5'-GYGGATTATGGTTTTGGTT-3'
Reverse: 5'-TTTTCTTTCRAAACAACATCCT-3'
Sequence for HOTAIR si-RNA
Forward: 5'-GAGGCGCUAAUUAAUUGAUTT-3'
Reverse: 5'-AUCAAUUAAUUAGCGCCUCTT-3'
HOTAIR expressing Sequence:
Forward: 5'-cCCGCTCGAGCCAGTTCTCAGGCGAGAGC-3';
Reverse : 5'-CGGGATCCTTTATATTCACCACATGTAAAACTT-3'
SAV1 Overexpression plasmid was purchased form Bio link company (Shanghai China)
